# Supplementary material for: Identification of Skin Lesions by Using Single-Step Multiframe Detector
Source: J Clin Med. 2021 Jan 4;10(1):144. doi: 10.3390/jcm10010144 (PMC7796252; doi:10.3390/jcm10010144)
Supplement: Supplementary file 1 [file jcm-10-00144-s001.pdf]

Journal of Clinical Medicine

Article

Identification of skin lesions by using single-step multiframe detector

Yu-Ping Hsiao [1,2], Chih-Wei Chiu [3], Chih-Wei Lu [4], Hong Thai Nguyen [3], Yu Sheng Tseng [3], Shang-Chin Hsieh [5,*] and Hsiang-Chen Wang [3,*]

1 Department of Dermatology, Chung Shan Medical University Hospital, No.110, Sec. 1, Jianguo N. Rd., South Dist., Taichung City 40201, Taiwan
2 Institute of Medicine, School of Medicine, Chung Shan Medical University, No.110, Sec. 1, Jianguo N. Rd., South Dist., Taichung City 40201, Taiwan
3 Department of Mechanical Engineering and Center for Innovative Research on Aging Society (CIRAS), National Chung Cheng University, 168, University Rd., Min Hsiung, Chia Yi 62102, Taiwan; gskyjob01@gmail.com (C.W.C.); lonesome310160@hotmail.com (Y.S.T.); nguyenhongthai194@gmail.com (H.T.N.)
4 Director of Technology Development, Apollo Medical Optics, Inc. (AMO), 2F., No. 43, Ln. 188, Ruiguang Rd., Neihu Dist., Taipei City 114, Taiwan; cwlu@mdamo.com (C.W.L.)
5 Department of Plastic Surgery, Kaohsiung Armed Forces General Hospital, 2, Zhongzheng 1st.Rd., Lingya District, Kaohsiung City 80284, Taiwan; sschin522@yahoo.com.tw (S.C.H.)
* Correspondence: sschin522@yahoo.com.tw (S.C.H.); hcwang@ccu.edu.tw (H.C.W.)
† These authors contributed equally to this work.

Received: date; Accepted: date; Published: date

S1. OCT images

By utilizing a single-crystal fiber light source, ApolloVue S100 provides high-axial resolution around one micron. Ti:sapphire crystal is a popular laser gain medium and also famous as the light source for high-resolution optical coherence tomography. By drawing the Ti:sapphire crystal into a fiber structure and pumping with laser diodes, broadband fluorescence emission with near-Gaussian line shape can be generated [S1]. Since the radiance is more than one order higher than conventional broadband spatially-incoherent light sources, FF-OCT imaging speed can also be improved with the compact and low-cost Ti:sapphire crystal fiber light source. On the other hand, the FF-OCT configuration is suitable for a high-resolution design due to the dynamic focusing method. In a high-NA (numerical aperture) OCT system, the short depth of focus range usually limits the depth scanning range to less than 100 μm but not due to the scattering of the sample. However; in the FF-OCT system, the focus plane is dynamically moved to match the coherence plane during the time-domain optical path scanning. The dynamic focusing scanning maintains the lateral resolution in the depth scanning range. Combining the single-crystal fiber light source and dynamic focus scanning, ApolloVue S100 (Fig. S1) can provide three-dimensional in-vivo cellular images for monitoring the pathologic features of tissue.

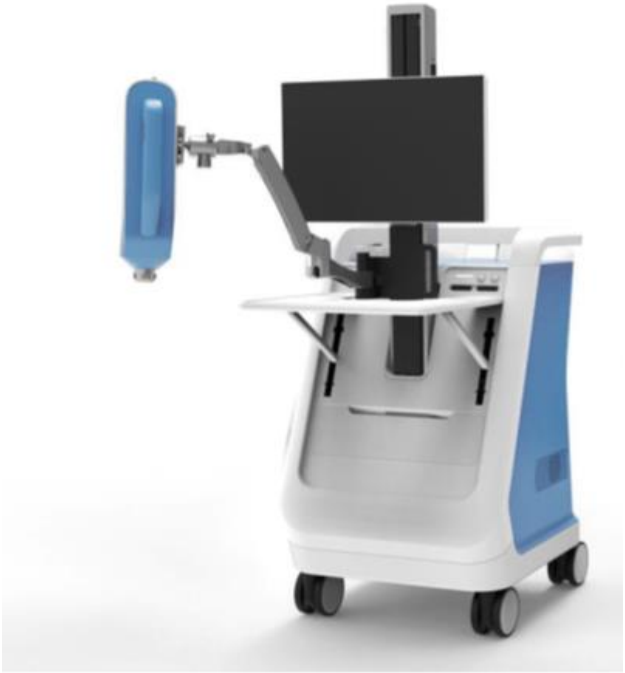

Figure S1. ApolloVue S100

For *in-vivo* measurement, the scanning speed is important for clinical efficiency to shorten the examining time and avoid motion blur of image. Medical devices based on reflectance confocal microscopy, two-photon microscopy and FF-OCT are commercially available for high-resolution *en-face* imaging in real time. However, in order to explore the cross-sectional structure of tissue, the transverse scanning scheme is not efficient. For many clinical applications, cross-sectional image can observe the distribution of tissue layers which is also similar to the direction of biopsy. The commercial Fourier-domain OCT could provide high-speed cross-sectional imaging, but all of them do not have adequate resolution to monitor skin diseases. The limitation of scanning speed for an FF-OCT system comes from the radiance of a light source and the frame rate of the camera. In this parallel detection scheme, conventional broadband light source with low spatial coherence is preferred because of lower cost and less coherent crosstalk [S2]. But since the radiance of spatially-incoherent light sources like incandescent lamps and light emitting diodes is relatively low, spatially-coherent and high-radiance light sources like supercontinuum sources are used in some high-speed application [S3]. Once the illumination is sufficient, the detection should also fast enough to receive all the back-scattered light from tissue. Since the frame rate of the camera is proportional to the number of pixels activated, the camera frame rate can be increased by setting a region of interest (ROI). An OCM for high-speed *en-face* imaging with a ROI is recently reported [S4].

In order to realize the cross-sectional scanning in the FF-OCT scheme, ApolloVue S100 shape the illumination to a line to increase the radiance and just active several lines of the two-dimensional camera to increase the frame rate. This method preserves a high-resolution feature of an FF-OCT but provides a cross-sectional scan with a decent speed for *in-vivo* measurement. The transverse scanning for *en-face* imaging is also contained in the ApolloVue S100 for laterally exploring the tissue. Compare to traditional full-field OCT or confocal microscopy, the cross-sectional image can be shown in real-time without reconstruction after whole volume is scanned. With a simple optical switch, user can switch between two modes to improve the efficiency of lesion examination and gather more structure information.

The FF-OCM setup can be breakdown into two parts. The first part is the illumination system consisted of the diode-pumped Ti:sapphire crystal fiber light source and the illumination optics. The second part is the imaging system consisted of a Mirau interferometer, a projection lens and a two-dimensional camera (Photonfocus MV1-D1024E-160). The core size of the crystal fiber is around 18 μm, and the broadband fluorescence emission was collected by an objective lens (NA=0.75). The

corresponding axial resolution is measured to be 1.3 μm in the tissue. Since the emitting area (about the core size of the crystal fiber) is small, the etendue of the crystal fiber light source is more than two orders smaller than conventional incandescent lamps and light emitting diodes.

The system configuration is shown in Fig. S2. For the ease of application, the fluorescence from the Ti:sapphire crystal fiber is coupled into a multimode fiber (Thorlabs FG105LCA) before entering the illumination optics. The illumination optics contains an achromatic collection lens and a second lens mounted on a dual-position slider (Thorlabs ELL6K). The system can be switched between *en face* mode and cross-sectional (B-scan) mode by changing the state of the slider. In the *en-face* mode, the second lens is a spherical field lens imaging the fiber tip to the rear focal plane of the objective lens, and the sample is illuminated with an oblique illumination scheme. In the B-scan mode, the second lens is switched to a cylindrical lens and the field of illumination (FOI) is in elliptical shape. With the Mirau configuration, the path-length scanning and dynamic focusing is simultaneously performed by translating the whole interferometer. The OCM images are then generated by demodulating the image sequence recorded by the camera. With a customized immersion-type objective lens (NA=0.8), the lateral resolution is about 1 μm. The immersion liquid was chosen to minimize the walk-off between the confocal and coherent gate to maintain the lateral resolution within the scanning range. An *en face* image is acquired with a frame rate of 11 Hz.

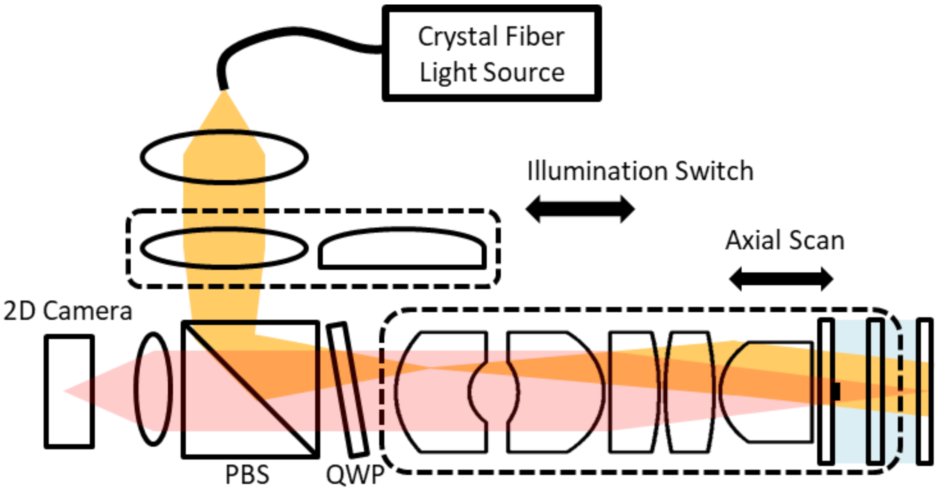

Figure S2. The schematic diagram of the Mirau-type OCM. PBS: polarization-sensitive beamsplitter. QWP: quarter-wave plate. Yellow ray: illumination ray. Pink ray: imaging ray.

While switching to the B-scan mode, the camera switches the ROI to a strip of the sensor to improve the frame rate. The motion of slider and the control of camera are synchronized so that the whole switching process is less than one second which is practical for clinical use. The number of lines is chosen so that the virtual slice thickness is a few microns, comparable to the typical thickness of histological sections. By activating multiple lines of pixels, closely adjacent B-scans can be acquired simultaneously, and the speckle noise in the image can be suppressed with spatial compounding [S5]. To maximize the radiant flux per pixel, the elliptical FOI shall match the shape of the strip ROI. With paraxial approximation and optical invariant, one can estimate the minimum width of illumination field $\phi_{FOI,min}$ (without losses) as:

$$\phi_{FOI,min} \cong (\phi_s \theta_s)/(n_o \theta_o) \quad \text{(s1)}$$

where n_o is the average refractive index in the objective space, and θ_o is the maximum marginal ray angle in the objective space, limited by the NA of the objective lens. The ϕ_s and θ_s is the width and emitting angle of the light source, and the product of them is the one-dimensional etendue. Since a smaller etendue is required to generate narrower FOI, the B-scan imaging speed and signal-to-noise ratio (SNR) is dependent on the radiance instead of total power of light source. With the high-radiance Ti:sapphire crystal fiber light source, the $\phi_{FOI,min}$ is in the order of tens of microns. Since the radiant flux per pixel is much higher in B-scan mode, the integration time of the

camera can be much reduced without loss of SNR. The integration time is one order less than in *en face* mode, and the axial scanning speed is 85 μm/second in this preliminary study.

AMO FF-OCT is a contact imaging system with a guiding system which provides wide-range real-time color imaging to guide OCT measurement on precise position. While a measurement starts, the cart can be moved to the patient and adjust the height by lifter which covers sitting and lying positions. Then, user can precisely align the probe to a lesion by moving the arm with the guiding video which is shown on monitor. Once the lesion is aligned, user can switch the system from guiding mode to OCT modes and perform OCT scans and save the images. After the scans, user can switch back guiding mode for next position. The modes can be selected through the buttons on the probe, the pedal or the computer. OCT images and the scanning positions on the guiding image will be recorded simultaneously for following up the lesion.

The OCT is a noninvasive optical imaging modality and has been widely used to diagnose ophthalmic diseases in the clinic [S6-S11]. With the use of an interferometry technique, the OCT can differentiate the back-scattered light from different layers within the sample and reconstruct the microstructure of a tissue. In this study, the ApolloVue S100 OCT system was used for the *in vivo* imaging of the microstructure of the skin and was based on the full-field OCT configuration [S12-S17]. The detailed experimental setup is shown in the Supplementary Information S1. Figure S3(a) is an image of the back of the hand of a patient with AD, in which the red box indicates the area with AD, while the blue box shows the patient's normal skin area. In Figure S3(b), a horizontal black line was evident at the position where the red arrow points, which is the transparent layer of the normal skin. Compared with the blue horizontal line marked in Figure S3(d), the black line fell on the same horizontal surface, and the stratum corneum was evenly distributed above the transparent layer. The skin lesions could be observed in Figure S3(c), in which the transparent layer is shown by the red arrow. Comparing the left and right sides of the blue horizontal lines in Figure S3(c) shows a drop on the right, which shows the stratum corneum of the diseased skin, and thickening was observed in the right area [S18]. Figure S4(a) is an image of the back of a patient with Pso, and the red box indicates the area with Pso. The blue box shows the patient's normal skin area. As shown in Figures S4(b) and S4(c), a vertical blue scale line was marked between the blue horizontal line and the skin surface. The height of the scale line represents the total thickness of each layer. Figure S4(b) shows the normal distribution of the stratum corneum, transparent layer, and granular layer in the normal skin area of the patient with Pso. Compared with Figure S4(c), the uppermost layer of the stratum corneum showed only a thickening, which squeezed the distribution of the lower layers. Therefore, the area pointed by the red arrow in Figure S4(c) was slightly squeezed by the development of the white area, which was selected by the dashed blue box as a slightly white image [S19]. Figure S5(a) is an image of the arm of a patient with skin lymphoma. The red box indicates the area with skin lymphoma cancer, while the blue box indicates the patient's normal skin area. Figure S5(b) shows the normal distribution of the stratum corneum, transparent layer, and granular layer in the normal skin area of the patient with skin lymphoma. The three blue vertical scale lines in Figure S5(c) indicate the different thicknesses of the stratum corneum of the diseased skin. Comparison of Figures S5(b) and S5(c) shows that the stratum corneum was abnormally thickened as shown by the three red arrows [S20].

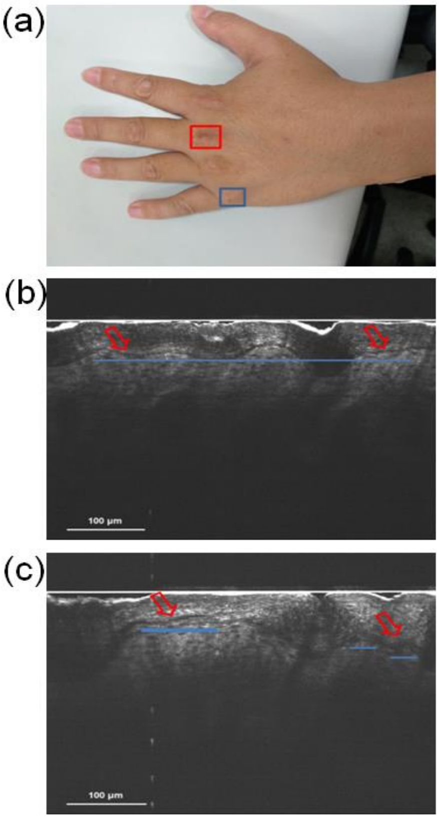

Figure S3. (a) Back of the hand of a patient with AD. The red box selects the area with AD, while the blue box selects the patient's normal skin area. OCT images of the (b) normal skin and (c) the area of onset AD.

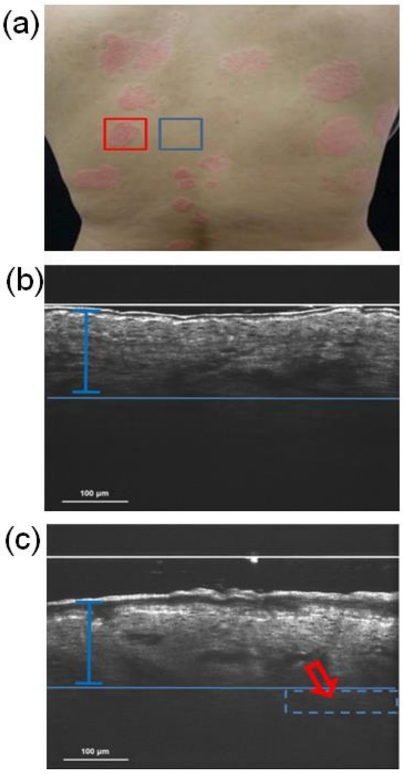

Figure S4. (a) Back of a patient with Pso. The red frame selects the area with Pso, while the blue frame selects the patient's normal skin area. OCT images of the (b) normal skin area and (c) the affected skin area.

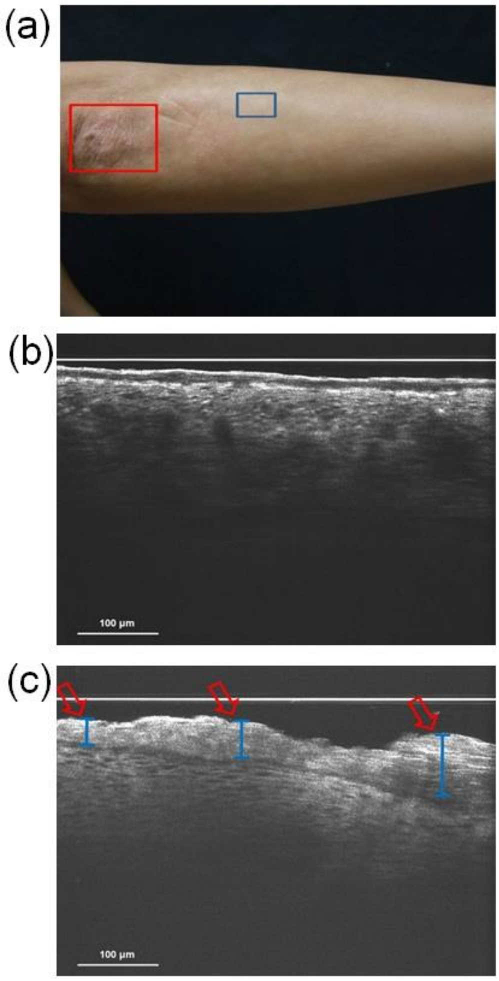

Figure S5. (a) Arm of a patient with skin lymphoma. The red box selects the area with skin lymphoma cancer, while the blue box circle selects the patient's normal skin area. OCT images of the (b) normal skin and (c) affected skin.

S2. Image data amplification

Due to the difference in the number of patients in each case, the number of images is uneven. We use data augmentation. Data augmentation can be used to teach the model about the invariance of images in the data [S21]. To a certain extent, the neural network can maintain the important symmetry, which can improve the performance of the neural network, and avoid overfitting, so that the model can achieve a better generalization effect, as shown in the Figure S6.

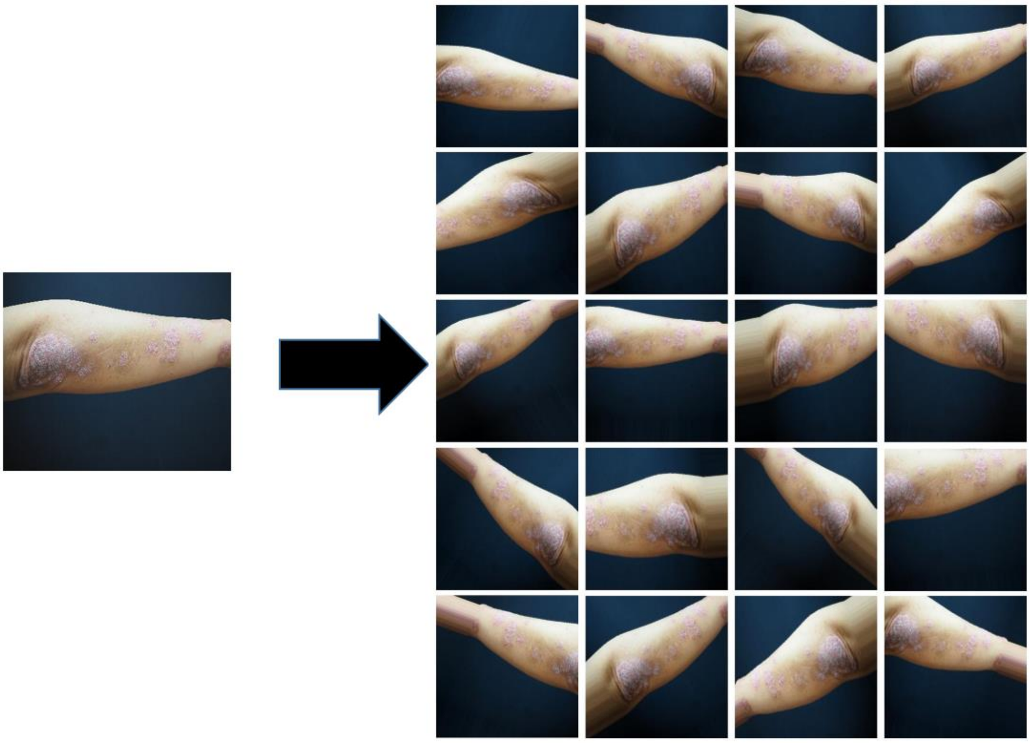

Figure S6. Image data amplification

S3. Single Shot MultiBox Detector

The SSD model is based on the VGG16 architecture and adds Conv4_3, Conv7, Conv8_2, Conv9_2, Conv10_2, Conv11_2 convolutional layers for image multi-scale feature extraction [S22]. The SSD model will set default boxes of different scales or aspect ratios for each target, and each pre-selection box will have an independent predicted value. These predicted values are mainly divided into two parts.

(1) Confidence of each category

The background that is not framed in the image is also regarded as a category. For example, if the detection target has c categories, the SSD actually needs to predict c+1 confidence levels, one of which refers to the part that does not contain the target or belongs to the background. The category with the highest confidence is the category to which the bounding box belongs, so the predicted bounding boxes are based on these preselected boxes.

(2) Positioning of the bounding box

In each feature map cell, what the model predicts is not the absolute coordinates of the bounding box in the image, but the offset between the bounding box and the preselected box. Each detected bounding box is relative to a preselection box, which does not exactly match the actual size of the object. Therefore, a zoom factor is needed to predict the size difference between the bounding box and the preselected box, and the zoom factor is used to calculate the true size of the bounding box in the image. Assume that the position of the preselection box is represented by d = $(d^{cx}, d^{cy}, d^{w}, d^{h})$. The corresponding bounding box position is represented by b = $(b^{cx}, b^{cy}, b^{w}, b^{h})$. The scaling factor is represented by l = $(l^{cx}, l^{cy}, l^{w}, l^{h})$. Where (cx, cy, w, h) respectively represent the center coordinates and width and height of the bounding box. The purpose of these assumptions is to make the bounding box match the preselection box, that is, b ≈ d. The method is to first align the center coordinates of the bounding box with the center coordinates of the preselection box. It means to "translate" the center point of the bounding box to the center point of the preselection box, as shown in equations s2 and s3. Then the size of the bounding box is "zoomed" to be close to the preselected box, such as equations s4 and s5. Through the above translation transformation and scale

scaling, the bounding box can be infinitely close to the preselected position. This method is called border regression.

$$b^{cx} = d^{w} l^{cx} + d^{cx} \quad \text{(s2)}$$

$$b^{cy} = d^{h} l^{cy} + d^{cy} \quad \text{(s3)}$$

$$b^{w} = d^{w} \exp(l^{w}) \quad \text{(s4)}$$

$$b^{h} = d^{h} \exp(l^{h}) \quad \text{(s5)}$$

In the process of training the model, we must first determine how the ground truth in the training image matches the preselection box. Here, the IOU value (Intersection-over-union, also known as Jaccard overlap) in the MultiBox is used to calculate the matching degree between the preselection box and the ground truth. Each ground truth box corresponds to a unique preselection box, and the bounding box corresponding to the preselection box will be responsible for predicting it. IOU is between 0 and 1. The larger the value, the higher the matching degree between the preselection box and the ground truth. In an ideal situation, the IOU of the prediction box and ground truth is 100%. Figure S7 is the flow chart of preselection box matching ground truth. There are two main matching principles:

(1) The first principle: For each ground truth in the picture, find the preselection box with the largest IOU, then the preselection box is matched with it, which can ensure that each ground truth must match a certain preselection box. The preselection box that matches the ground truth is positive samples. On the contrary, if a preselection box does not match any ground truth, it matches the background, and it is a negative sample [S23].

(2) The second principle: For the remaining unmatched pre-selection boxes, if the IOU value of a certain ground truth is greater than a certain threshold (usually 0.5), then the remaining pre-selection boxes are also matched with the ground truth, which means a certain ground truth may match multiple preselection boxes. This is possible, because a preselection box can only match one ground truth. If multiple ground truths and a certain preselection box IOU are greater than the threshold, then the preselection box is only preselected with the largest IOU Box to match.

The second principle must be carried out after the first principle. First, it is necessary to ensure that a certain ground truth must have a preselection box to match it. Due to the large number of preselected boxes, the maximum IOU of a certain ground truth must be greater than the threshold.

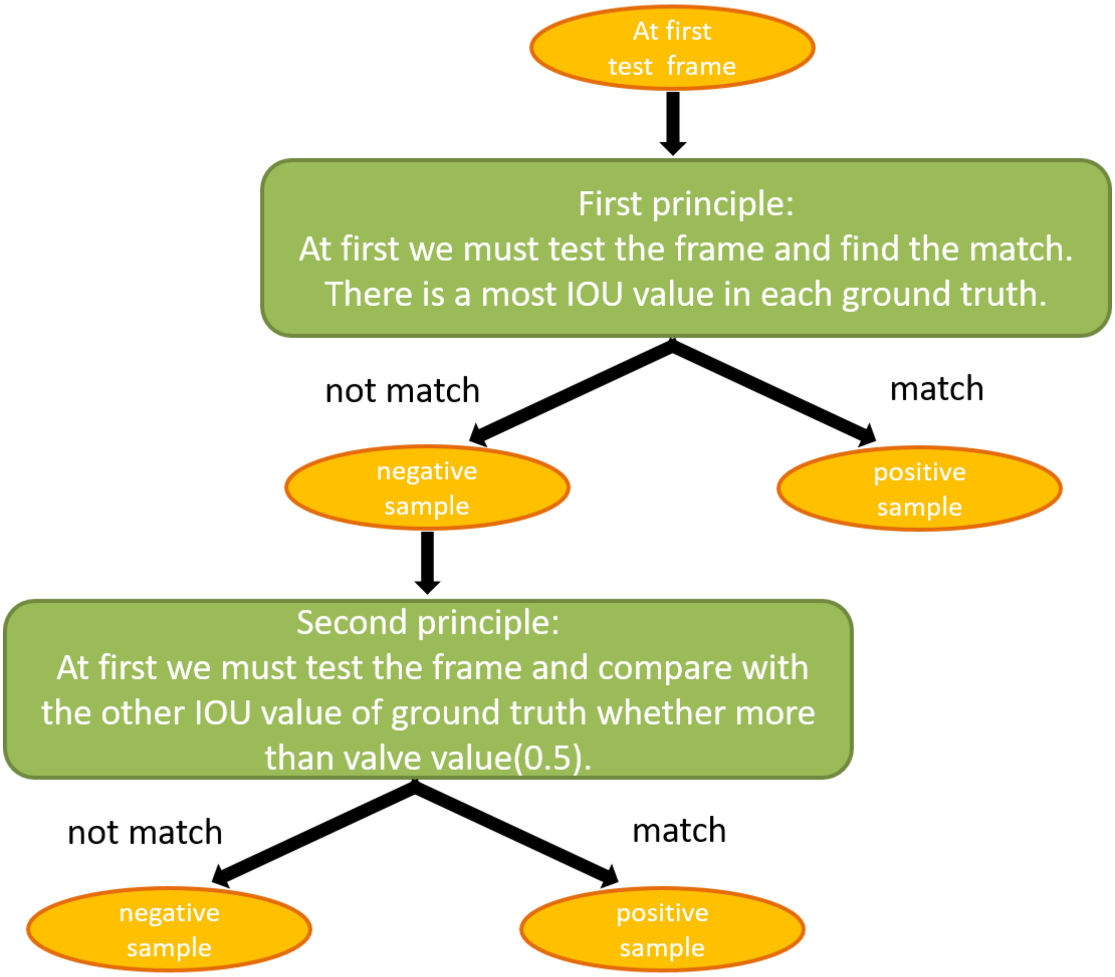

Figure S7. Preselection box and real matching flow chart

The SSD loss function is divided into location error (localization loss, loc) and confidence error (confidence loss, conf).

(1) localization loss, loc

It is used to calculate the error between the preselection box and the ground truth position information. The definition is as shown in equation S6, and the smooth L1 loss is used [S24]:

$$L_{loc}(x,l,g)=\sum_{i\in Pos}^{N}\sum_{m\in\{cx,cy,w,h\}} x_{ij}^{k}\,\mathrm{smooth}_{\mathrm{L1}}(l_i^m-\hat{g}_j^m) \qquad \text{(S6)}$$

The preselection box, like ground truth, uses the border regression mentioned earlier to match, but because what needs to be calculated now is the position error of the two. So the calculation result is no longer the position of the ground truth, but the offset between the preselection box and the ground truth.In formula S6, suppose that the 4 values of the prediction result output are the predicted value of the preselection box $(l_i^{cx}, l_i^{cy}, l_i^{w}, l_i^{h})$. The purpose is to first calculate the offset $(\tilde{g}_j^{cx}, \tilde{g}_j^{cy}, \tilde{g}_j^{w}, \tilde{g}_j^{h})$ between the preselected boxes $d_i = (d_i^{cx}, d_i^{cy}, d_i^{w}, d_i^{h})$ and ground truth $g_j = (g_j^{cx}, g_j^{cy}, g_j^{w}, g_j^{h})$. Then use the smooth L1 of formula S6 to calculate the error between the two, such as formula S7 and S8. Using the "first translation" of boundary regression, the formula can be written in the form of equations S2 and S3. But the goal is to calculate the offset, so the formula is rewritten into the form of formula S7 and S8. In a similar way, formulas S9 and S10 can be obtained, which convert the exponential function into a logarithmic function to ensure that the value in log is greater than zero.

$$\hat{g}_j^{cx} = (g_j^{cx} - d_i^{cx})\,/d_i^{w} \qquad \text{(S7)}$$
$$\hat{g}_j^{cy} = (g_j^{cy} - d_i^{cy})\,/d_i^{h} \qquad \text{(S8)}$$

$$\hat{g}_j^w = \log\left(\frac{g_j^w}{d_i^w}\right) \quad (S9)$$

$$\hat{g}_j^h = \log\left(\frac{g_j^h}{d_i^h}\right) \quad (S10)$$

$$\text{smooth}_{L1}(x) = \begin{cases} 0.5x^2 & if\ |x| < 1 \\ |x| - 0.5 & otherwise \end{cases} \quad (S11)$$

(2) confidence loss, (conf)

The definition is as shown in formula S11 using softmax loss:

$$L_{conf}(x,c) = -\sum_{i\in Pos}^{N} x_{ij}^k\, log(\hat{c}_i^p) - \sum_{i\in Neg} log(\hat{c}_i^o) \quad (S12)$$

$$,\quad \hat{c}_i^p = \frac{\exp(c_i^p)}{\sum_p \exp(c_i^p)}$$

In formula S12, $\hat{c}_i^p$ represents the confidence level of the target of p categories in the i-th preselection box. The term "$-\sum_{i\in Pos}^{N} x_{ij}^k\, log(\hat{c}_i^p)$" indicates that there is a confidence score of the positive sample that matches the ground truth. Multiply the probability of a target by the confidence of the target to get the confidence score of the target. So it takes into account the probability of the target and the target category. The term "$-\sum_{i\in Neg} log(\hat{c}_i^o)$" indicates the confidence of the negative sample that does not match the ground truth. In SSD, the background is a special category, so the negative sample will match the background, then the target category will not be considered. If you only consider whether the target exists, add the confidence of the positive and negative samples to get the confidence loss of formula S12.

After understanding the two error categories, you can define the loss function of the SSD. The loss function is defined as the weighted sum of the location error (localization loss, loc) and the confidence error (confidence loss, conf) [S25], and the calculation is as shown in equation S13.

$$L(x,c,l,g) = \frac{1}{N}(\alpha L_{conf}(x,c) + \alpha L_{loc}(x,l,g)) \quad (S13)$$

where N is the number of positive samples of the preselection box. Here $x_{ij}^p \in \{1,0\}$ is an indicator parameter.When x_{ij}^p =1, it means that the i-th preselection box matches the j-th ground truth, and the category of ground truth is p. c is the category confidence prediction value, l is the location prediction value of the bounding box corresponding to the preselection box, and g is the location parameter of the ground truth. This definition meets the requirements of model training.

The key feature of the proposed model is its at least one order of magnitude higher number of a priori boxes with different positions, scales, and aspect ratios than existing methods and the output of multiscale convolutional bounding boxes by using multiple feature maps [S26, S27]. These characteristics indicate that the model needed not learn too complex image boundary features, and the model could be efficiently trained to place a bounding box on the affected space. All the layers (filters) of the SSD were finetuned by using random gradient descent.

The model setting can select multiple rectangular a priori boxes of different sizes and positions, but the generated prediction results by the model often only need a few bounding boxes to match the ground truth. Thus, the SSD needs to be able to match or eliminate redundant a priori boxes. The matching principle between a priori box and ground truth are defined as follows. First, how the ground truth in the training image matched the prior box was determined. Here, the IOU value was used to calculate the degree of matching between the prior box and ground truth [S28]. This process ensured that each ground truth corresponded to a unique a priori box, and the bounding box corresponding to the matching a priori box would be responsible for its prediction. The IOU value was between 0 and 1. The larger the value, the higher the matching degree between the a priori box and the ground truth would be. In an ideal situation, the IOU of the prediction box and the ground truth is 100%. Figure S8 shows a schematic diagram of the principle of a priori box matching with the ground truth. The green box is the ground truth, the multiple red boxes are the a priori boxes, and the blue box is the bounding box that finally matches the ground truth. As shown in Figure S8(a), the SSD formed multiple a priori boxes to match the ground truth. Two main matching principles were considered. The first principle was to find the a priori box with the largest IOU for each ground truth

in the picture. The matching of the check box could ensure that each ground truth should match a certain a priori box, as shown in Figure S8(d). The a priori box that matched the ground truth was called a positive sample. After identification as the positive sample, the box became a bounding box. However, if a prior box did not match any ground truth, then the a priori box could only match the background and was considered as a negative sample [Figure S8(b)]. Very few ground truth areas but many a priori boxes were found in the images. If only the first principle was matched, many a priori boxes would be considered as negative samples, and the ratio of positive and negative samples would be extremely unbalanced. Thus, the second principle was needed. For the remaining unmatched a priori boxes, if the IOU value of a ground truth was greater than a certain threshold (usually 0.5), then the a priori box was also matched with the ground truth, as shown in Figure S8(c). This phenomenon indicates that a certain ground truth may match multiple prior boxes. Conversely, an a priori box could only match one ground truth. If multiple ground truths and a certain a priori box IOU were greater than the threshold, then the a priori box was only matched with the a priori box with the largest IOU. Figure S8(e) shows that the final output bounding box (the SSD final prediction result output) also complies with the first and second principles. After using the SSD to learn the training image set, 300 independent test images were used to evaluate the performance of the trained model. When the model detector detected skin lesions from the input data of the test image, the disease name (Pso, AD, MF, and Normal) and a rectangular frame in the skin image were displayed to surround the lesion area and analyze the evaluation results with the following indicators: recall, precision, and F1-score.

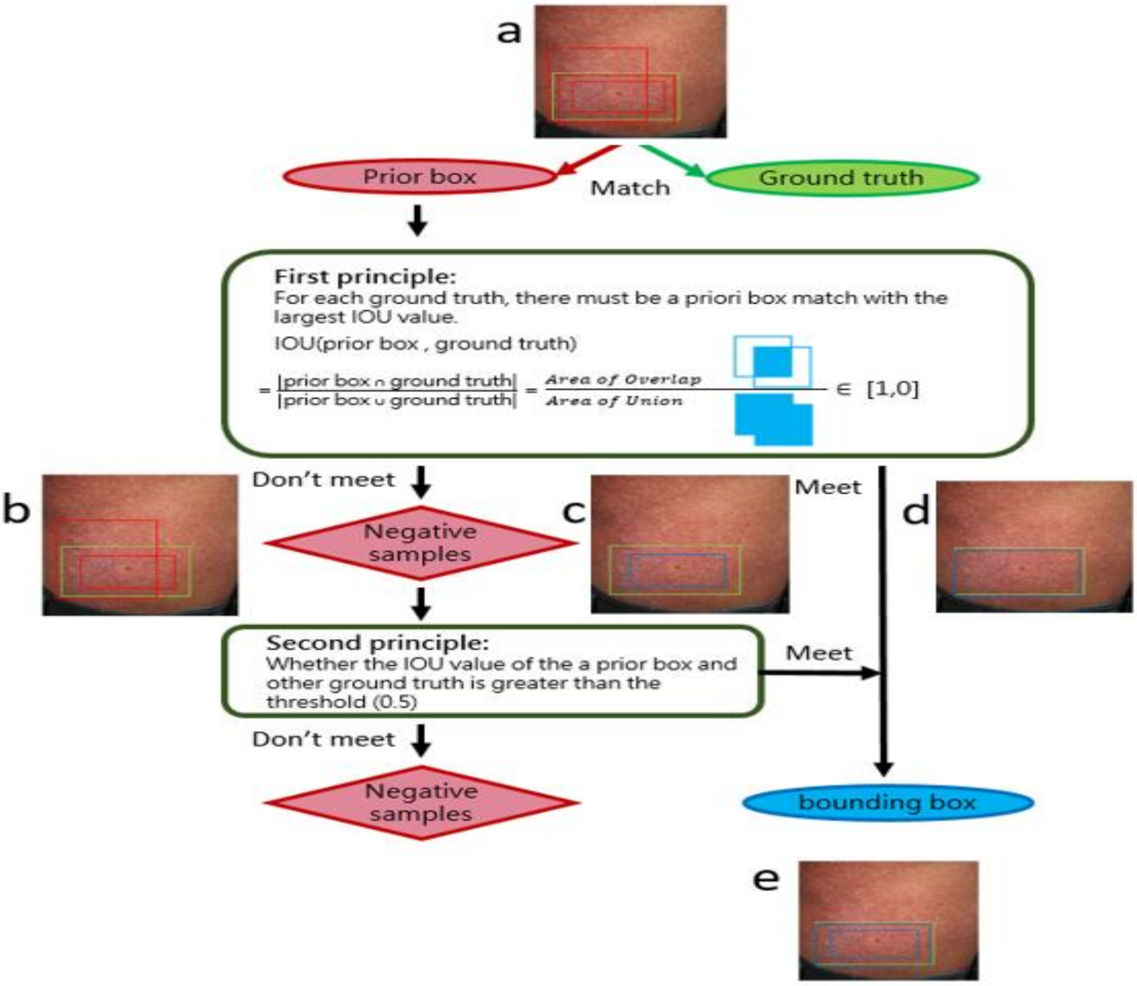

Figure S8. A schematic diagram of the principle of a priori box matching ground truth. The green box in the skin image is the ground truth, the multiple red boxes are the a priori boxes, and the blue box is the bounding box that finally matches the ground truth. Figure S8(a) shows the matching of multiple prior boxes with the ground truth, S8(b) shows the negative samples that do not meet the first principle during the matching process, and S8(c) shows the positive samples that meet the first principle and will become a bounding box. Figure S8(d) is a positive sample that does not conform to the first principle but conforms to the second principle. This box will also subsequently become a bounding box. Figure S8(e) is the final output bounding box, which conforms to the first and second principles, and the final output of the SSD prediction result.

References

[S1]. Wang, S.C., Yang, T.I., Jheng, D.Y., Hsu, C.Y., Yang, T.T., Ho, T.S., Huang, S.L. Broadband and high-brightness light source: glass-clad Ti: sapphire crystal fiber. *Optics letters* **2015**, *40*, 5594-5597.

[S2]. Karamata, B., Lambelet, P., Laubscher, M., Salathé, R. P., Lasser, T. (2004). Spatially incoherent illumination as a mechanism for cross-talk suppression in wide-field optical coherence tomography. *Optics letters* **2004**, *29*, 736-738.

[S3]. Dubois, A., Levecq, O., Azimani, H., Siret, D., Barut, A., Suppa, M., ... & Perrot, J. L. Line-field confocal optical coherence tomography for high-resolution noninvasive imaging of skin tumors. *Journal of biomedical optic*s **2018**, *23*, 106007.

[S4]. Ogien, J., Dubois, A. A compact high-speed full-field optical coherence microscope for high-resolution in vivo skin imaging. *Journal of biophotonics* **2019**, *12*, e201800208.

[S5]. Schmitt, J. M., Xiang, S. H., Yung, K. M. Speckle in optical coherence tomography. *Journal of biomedical optics* **1999**, *4*, 95-105.

[S6]. Wolff, B.; Matet, A.; Vasseur, V.; Sahel, J.-A.; Mauget-Faÿsse, M. *En face* OCT imaging for the diagnosis of outer retinal tubulations in age-related macular degeneration. *Journal of ophthalmology* **2012**, *2012*.

[S7]. Kim, K.E.; Kim, J.M.; Song, J.E.; Kee, C.; Han, J.C.; Hyun, S.H. Development and Validation of a Deep Learning System for Diagnosing Glaucoma Using Optical Coherence Tomography. *Journal of Clinical Medicine* **2020**, *9*, 2167.

[S8]. Liu, F.; Saul, A.B.; Pichavaram, P.; Xu, Z.; Rudraraju, M.; Somanath, P.R.; Smith, S.B.; Caldwell, R.B.; Narayanan, S.P. Pharmacological inhibition of spermine oxidase reduces neurodegeneration and improves retinal function in diabetic mice. *Journal of Clinical Medicine* **2020**, *9*, 340.

[S9]. Iovino, C.; Pellegrini, M.; Bernabei, F.; Borrelli, E.; Sacconi, R.; Govetto, A.; Vagge, A.; Di Zazzo, A.; Forlini, M.; Finocchio, L. Choroidal vascularity index: an in-depth analysis of this novel optical coherence tomography parameter. *Journal of Clinical Medicine* **2020**, *9*, 595.

[S10]. Yao, H.-Y.; Tseng, K.-W.; Nguyen, H.-T.; Kuo, C.-T.; Wang, H.-C. Hyperspectral Ophthalmoscope Images for the Diagnosis of Diabetic Retinopathy Stage. *Journal of Clinical Medicine* **2020**, *9*, 1613.

[S11]. Parra-Blesa, A.; Sanchez-Alberca, A.; Garcia-Medina, J.J. Clinical-Evolutionary Staging System of Primary Open-Angle Glaucoma Using Optical Coherence Tomography. *Journal of Clinical Medicine* **2020**, *9*, 1530.

[S12]. Dubois, A.; Grieve, K.; Moneron, G.; Lecaque, R.; Vabre, L.; Boccara, C. Ultrahigh-resolution full-field optical coherence tomography. *Applied optics* **2004**, *43*, 2874-2883.

[S13]. Wang, S.-C.; Yang, T.-I.; Jheng, D.-Y.; Hsu, C.-Y.; Yang, T.-T.; Ho, T.-S.; Huang, S.-L. Broadband and high-brightness light source: glass-clad Ti: sapphire crystal fiber. *Optics letters* **2015**, *40*, 5594-5597.

[S14]. Karamata, B.; Lambelet, P.; Laubscher, M.; Salathé, R.; Lasser, T. Spatially incoherent illumination as a mechanism for cross-talk suppression in wide-field optical coherence tomography. *Optics letters* **2004**, *29*, 736-738.

[S15]. Dubois, A.; Levecq, O.; Azimani, H.; Siret, D.; Barut, A.; Suppa, M.; Del Marmol, V.; Malvehy, J.; Cinotti, E.; Rubegni, P. Line-field confocal optical coherence tomography for high-resolution noninvasive imaging of skin tumors. *Journal of biomedical optics* **2018**, *23*, 106007.

[S16]. Ogien, J.; Dubois, A. A compact high-speed full-field optical coherence microscope for high-resolution in vivo skin imaging. *Journal of biophotonics* **2019**, *12*, e201800208.

[S17]. Schmitt, J.M.; Xiang, S.; Yung, K.M. Speckle in optical coherence tomography. *Journal of biomedical optics* **1999**, *4*, 95-105.

[S18]. Byers, R.A.; Maiti, R.; Danby, S.G.; Pang, E.J.; Mitchell, B.; Carré, M.J.; Lewis, R.; Cork, M.J.; Matcher, S.J. Sub-clinical assessment of atopic dermatitis severity using angiographic optical coherence tomography. *Biomedical optics express* **2018**, *9*, 2001-2017.

[S19]. Egawa, M.; Kunizawa, N.; Hirao, T.; Yamamoto, T.; Sakamoto, K.; Terui, T.; Tagami, H. In vivo characterization of the structure and components of lesional psoriatic skin from the observation with Raman spectroscopy and optical coherence tomography: a pilot study. *Journal of dermatological science* **2010**, *57*, 66-69.

[S20]. Ring, H.C.; Stamp, I.M.H.; Jemec, G.B. Imaging cutaneous T-Cell lymphoma with optical coherence tomography. *Case Reports in Dermatology* **2012**, *4*, 139-143.

[S21]. Shorten, C., Khoshgoftaar, T. M. A survey on image data augmentation for deep learning. *Journal of Big Data* **2019**, *6*, 60.

[S22]. Simonyan, K., Zisserman, A. Very deep convolutional networks for large-scale image recognition. *International Conference on Learning Representations* **2015**, 1-14.

[S23]. Uijlings, J. R., Van De Sande, K. E., Gevers, T., Smeulders, A. W. Selective search for object recognition. *International journal of computer vision* **2013**, *104*, 154-171.

[S24]. Szegedy, C., Reed, S., Erhan, D., Anguelov, D., Ioffe, S. Scalable, high-quality object detection. *arXiv preprint arXiv* **2014**, 1412-1441.

[S25]. Girshick, R. Fast R-CNN. In *Proceedings of the IEEE international conference on computer vision* **2015**, 1440-1448.

[S26]. Liu, W.; Anguelov, D.; Erhan, D.; Szegedy, C.; Reed, S.; Fu, C.-Y.; Berg, A.C. In Ssd: Single shot multibox detector, European conference on computer vision, Springer: 2016, 21-37.

[S27]. Uijlings, J.R.; Van De Sande, K.E.; Gevers, T.; Smeulders, A.W. Selective search for object recognition. International journal of computer vision 2013, 104, 154-171.

[S28]. Simonyan, K.; Zisserman, A. Very deep convolutional networks for large-scale image recognition. arXiv preprint arXiv 2014.
